# Perineal resuturing versus expectant management following vaginal delivery complicated by a dehisced wound (PREVIEW): a nested qualitative study

**DOI:** 10.1136/bmjopen-2016-013008

**Published:** 2017-02-10

**Authors:** L Dudley, C Kettle, J Waterfield, Khaled M K Ismail

**Affiliations:** 1 The Maternity Centre, Royal Stoke, University Hospitals of North Midlands, Staffordshire, UK; 2 Faculty of Health Sciences, Staffordshire University, Stafford, UK; 3 School of Health and Rehabilitation and Institute for Primary Care and Health Sciences, Keele University, Staffordshire, UK; 4 Institute of Metabolism and Systems Research, College of Medical and Dental Sciences, University of Birmingham, Birmingham, UK

**Keywords:** Postnatal, Perineum, Dehiscence, Womens experience

## Abstract

**Objective:**

To explore women's lived experiences of a dehisced perineal wound following childbirth and how they felt participating in a pilot and feasibility randomised controlled trial (RCT).

**Design:**

A nested qualitative study using semistructured interviews, underpinned by descriptive phenomenology.

**Participants and setting:**

A purposive sample of six women at 6–9 months postnatal who participated in the RCT were interviewed in their own homes.

**Results:**

Following Giorgi's analytical framework the verbatim transcripts were analysed for key themes. Women's lived experiences revealed 4 emerging themes: (1) Physical impact, with sub-themes focusing upon avoiding infection, perineal pain and the impact of the wound dehiscence upon daily activities; (2) Psychosocial impact, with sub-themes of denial, sense of failure or self-blame, fear, isolation and altered body image; (3) Sexual impact; and (4) Satisfaction with wound healing. A fifth theme ‘participating in the RCT’ was ‘a priori’ with sub-themes centred upon understanding the randomisation process, completing the trial questionnaires, attending for hospital appointments and acceptability of the treatment options.

**Conclusions:**

To the best of our knowledge, this is the first qualitative study to grant women the opportunity to voice their personal experiences of a dehisced perineal wound and their views on the management offered. The powerful testimonies presented disclose the extent of morbidity experienced while also revealing a strong preference for a treatment option.

**Trial registration number:**

ISRCTN05754020; results.

**Strengths and limitations of this study**

- This nested qualitative study complements the data obtained from the PREVIEW pilot randomised controlled trial (RCT) adding a degree of comprehensiveness and richness to the whole study.
- Lessons learnt and knowledge gained will be pivotal to inform the design of the future definitive study and towards providing much needed answers towards the efficacy of the interventions.
- Only RCT participants were interviewed; including non-randomised women who fulfilled eligibility criteria may have proven beneficial towards the planning of the definitive study.
- Owing to geographical locations of the recruiting sites for the pilot RCT, five of the six women interviewed were from one organisation and therefore may not truly reflect women's experiences of participating in research at the other sites.
- The free text annotations in the RCT questionnaires do however suggest that women's experiences of taking part in the RCT were very positive, demonstrating the benefits of the mixed methodological approach.

## Introduction

Perineal trauma affects a vast amount of women worldwide with more than 350 000 women requiring suturing of a spontaneous tear or episiotomy in the UK per year.^1^ Given that the postpartum management of perineal trauma including the prevention of wound infection and assessing wound healing are core components of routine maternity care,^2^ ^3^ there is limited research evidence available on the women's experiences of the management and consequences of perineal wound infection and dehiscence.

A retrospective audit by Johnson and colleagues^4^ in 2012 suggested that 1 in 10 women who sustained a perineal tear at vaginal delivery that required suturing developed perineal wound infection. The authors defined wound infection as the presence of any two of the following markers: perineal pain, wound dehiscence or purulent vaginal discharge. The majority of these wounds will dehisce in the first 7–14 days following childbirth.^5–7^ However, robust systems to track wound dehiscence following hospital discharge are lacking and has led to the wide disparity of prevalence from 0.59%^8^ to 13.5%.^9^

Perineal wound dehiscence is associated with major physical, psychological and social problems and can pose a serious threat to the general well-being and quality of life of the new mother. Maternal morbidity centres on persistent pain and discomfort at the perineal wound site; urinary retention, defaecation problems, dyspareunia and psychological and psychosexual issues as a result of embarrassment and altered body image.^3^ ^10^ Furthermore, the mother/baby relationship may become affected and, indeed, can have a negative impact on the establishment of breast feeding.^11^

The literature exploring women's views of living with perineal trauma is sparse. However, there is more recent evidence^12–14^ which builds on earlier qualitative research,^15–17^ suggesting a renewed interest in this phenomena. While these qualitative studies have focused primarily on women's experiences of perineal trauma following Obstetric Anal Sphincter Injuries, the commonalities of both the physical and psychosocial findings of these studies have the potential to be applicable to women who sustain perineal wound dehiscence. Reading the women's unique accounts of living with the consequences of perineal trauma, clearly demonstrates the magnitude of physical, psychosocial and sexual morbidity they have experienced in the short and long term. We are not aware of any primary qualitative studies devoted to investigate women's experiences of perineal wound dehiscence. Therefore, the main objectives of the qualitative component of PREVIEW were: to explore women's thoughts regarding their experiences of a dehisced perineal wound; explore their views about being involved in a pilot RCT^18^ testing different management options for this complication and their acceptability of these choices.

## Methods/design

It is widely accepted that phenomenology has a great deal to offer health research as it provides the perspectives of those receiving the services (resuturing or expectancy in PREVIEW) which might open up understandings that may not be available through other methods.^19–22^

This qualitative study followed a descriptive phenomenological approach based on the work of Husserl E^23^ using semistructured interviews to answer the research questions.

Describing women's personal experiences underpinned this phase of the study, hence the need for the researcher to set aside any prejudgements and open any interview with an unbiased, receptive presence.^24^ As the researcher (LD) had neither a detailed professional knowledge of the most appropriate way to manage a dehisced perineal wound and no personal experience of childbirth the descriptive approach of Husserlian phenememology^24^ ^25^ was deemed the most appropriate to adopt. The researcher (LD) conducting the interviews chose to consciously explore her personal attitudes and beliefs towards the management of perineal wound dehiscence by the use of a reflective journal. This reflexivity as acknowledged by others then facilitated the evaluation of oneself, including how this may have influenced question phrasing, data collection and analysis, while also providing a verifiable audit trail of the research process,^22^ ^26^ ^27^ ultimately thereby enhancing the ethical and methodological rigour to the study.^28^

### Study population and sample size

The study population comprised a purposive sample of women who had participated in the PREVIEW pilot RCT to ensure that both arms of the study were equally represented. Based on guidance for phenomenological research, a sample size of between 6 and 12 participants was considered appropriate and should develop theoretical representation.^29^ ^30^ Guest and colleagues^31^ assert that if the sample is homogeneous and the experience under investigation is common among the population, smaller sample sizes are likely to be sufficient to achieve thematic saturation.

Interviews continued until data saturation was reached; identified when no new information was being revealed.^32^ Both the interviewer and a member of the qualitative research support team confirmed data saturation. The final sample determined by data saturation at interview was six (three from the resuturing arm and three managed by expectancy).

Women who indicated on the consent form for the RCT that they could be contacted to take part in the qualitative study were provided with a study information leaflet. Additional written consent for the interviews was obtained from women who agreed to participate. A choice of venue was offered for the interviews, but all chose to be interviewed in their own home.

### Data collection for the interviews

Interviews were conducted by LD using a semistructured interview schedule and digitally recorded with permission. Content validity for the interview guide was gained by a review of the literature, clinical experience of both the research team and fellow midwifery and obstetric colleagues and two patient representatives.

### Data analysis

Following a critical evaluation of the commonly referred to frameworks for the analysis of qualitative data using a Husserlian phenomenological approach^33–36^ a decision was made to follow Giorgi's analytical framework.^36^

A professional company was used for interview transcription. The transcripts were subsequently annotated in parts, highlighting laughter or expressions that were not detailed in the professional transcript that may affect the interpretation of the transcribed text.^37^

Following completion of stage 1 of Giorgi's method of data analysis, (reading all of the interview material to obtain a ‘sense of the whole’) the ‘one sheet of paper approach’ (OSOP) described by Ziebland and McPherson^37^ was applied. This necessitated the use of a large single sheet of paper (A3 size) to extract all the various issues raised by the women interviewed. Each extract was then represented by the woman's identification code 1–6, the sequence order of the interviews. The process was continued until all issues were noted on the paper. Commonalities appearing in the data (stage 2 of Giorgi's analysis) were then grouped into broader themes and subthemes. The relevance of each of these themes in relation to the phenomenon being investigated (dehisced perineal wounds) was then described (stage 3 of Giorgi's analysis). A sample of transcripts were coded and analysed independently by two researchers and the emerging themes discussed to ensure reliability. Incorporating this level of objectivity increased the credibility to the analysis phase of this study.

## Results

The individual characteristics of the six women interviewed are provided in table 1 (names have been changed to ensure confidentiality).

**Table 1:** Characteristics of the six women interviewed

Characteristics	Sue 1	Nicola |2	Diane 3	Fiona 4	Jenny 5	Cathy 6
Age	38	23	20	29	27	28
Ethnicity	White	White	White	White	White	White
Relationship status	Married	Cohabiting	Cohabiting	Married	Cohabiting	Cohabiting
Employment	Yes	Yes	No	Yes	Yes	Yes
1st vaginal delivery	No	Yes	Yes	No	Yes	Yes
Previous perineal trauma	Yes	NA	NA	Yes	NA	NA
Previous dehisced perineal wound	Yes	NA	NA	No	NA	NA
Mode of delivery	Normal	Normal	Forceps*	Normal	Forceps*	Ventouse*
RCT allocation R=resutured E=expectancy	R	E	R	E	R	E
Months postnatal	8	7	6	7	9	8
Length of interview (minutes)	29	18	23	18	26	40

*Forceps and Ventouse delivery are classified as operative vaginal deliveries.

**A**ll wounds dehisced within the first postnatal week.

NA, not applicable; RCT, randomised controlled trial.

**Figure 1:**
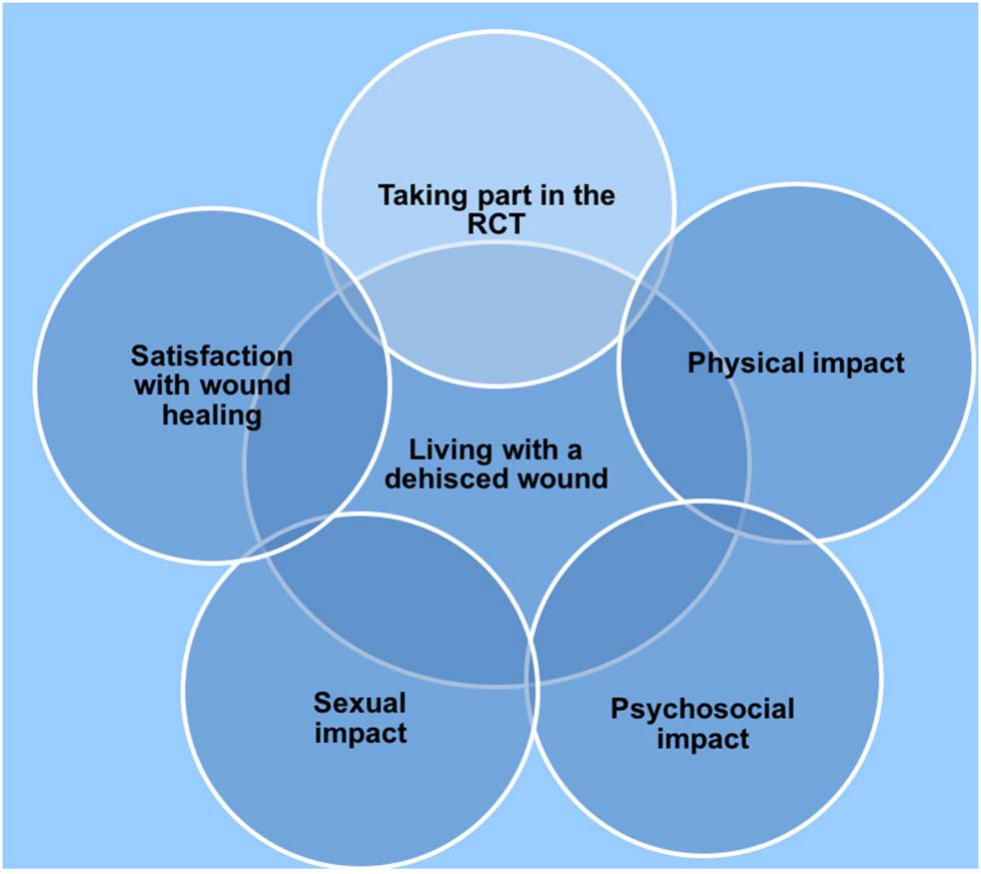
The five main themes obtained from six interviews with women who participated in the RCT (2011–2013). RCT, randomised controlled trial.

Five main themes (figure 1) supported by subthemes were identified. Four of the five themes were emergent and represented the experiences of all six women interviewed, these were:

- *Physical impact* with subthemes focusing on avoiding infection, perineal pain and the impact of the wound dehiscence on daily activities
- *Psychosocial impact* with subthemes of denial, sense of failure or self-blame, fear, isolation and altered body image
- Sexual impact
- Satisfaction with healing.

A fifth theme *participating in the RCT* was ‘a priori’, a term acknowledged as being derived from the characteristics of the phenomenon (dehisced perineal wounds) being studied.^38^ This ‘a priori’ theme is particularly relevant towards planning for the definitive study and establishing if the intervention was acceptable to women. Subthemes centred on understanding the randomisation process, completing the trial questionnaires, attending hospital appointments and acceptability of the treatment options.

### Theme 1: physical impact of perineal wound dehiscence

This theme describes the physical impact of the dehisced perineal wound and reflects on the descriptive words women used to express the type of pain they were experiencing and how this affected their activities of daily living. This theme also captures the concerns women have relating to infection and wound healing.

#### Subtheme: perineal pain

Perineal pain was one of the first areas raised by all women when they were asked to remember how they felt when their wound had broken down. The women used various terms to describe the intensity and depth of their pain for instance:

I thought I was dying {laughter}, it really hurt that much. (Nicola)

I was very sore. It was terrible to be honest. (Jenny)

It was petrifying. (Cathy)

When you are examined in the hospital and you took out the stitch … that was horrific. (Sue)

#### Subtheme: the impact of the wound dehiscence on daily activities

While the descriptors of pain were emotive, the impact of this pain on the women themselves and their families became clearly evident as the extracts below demonstrate when they started to talk about how the pain affected their daily living activities.

I contemplated putting him on the bottle, just because I'd had enough really and I wanted one of the pains to go away. (Sue)

I was quite surprised how much it did affect me to be honest because I thought, oh it will be alright, it will heal…but no, I was very sore…. so I really struggled once it happened. It was stopping me from doing simple things … I mean, even in the night I couldn't get up with him, do you know what I mean? And it's like I couldn't sit on the bed to feed him. I could hardly sit down. It was terrible, to be honest. When I was sitting downstairs, I was fine because I have the arm of the chair to lean on because I could only sit one-sided, but at night, when I have to sit on the end of the bed, I couldn't… I couldn't sit on the end of the bed. So, yeah, my partner had to do all the night feeding. (Jenny)

#### Subtheme: avoiding infection

Most of the women expressed concerns about infection and measures they took towards avoiding infection, particularly Jenny and Diane as the following extracts clearly illustrate.

I was worried about infection because of where it is and it was quite deep the wound…yeah, it was like so many centimetres, [demonstrates the depth with hands] … so yeah, I spent the night…hours in the shower … I was in the shower all the time, I just like wanted to make sure I didn't get any infection or anything … I tried to keep it clean. I was in the shower like, loads of times a day. (Jenny)

Diane, whose wound had completely dehisced, paused several times as she struggled with her emotions.

I think it was due to infection that it broke down so much. I was looking at them every day, to clean it. I could tell then and I got my mum to look at them as well. Because it didn't feel like it was, it was healing, and because I was so stressed and I didn't really want to go to the doctors anymore, I don't know. I was just…because I was feeling them as well just to see like how deep it was and I don't know … I don't know … it was really…rather I don't really know.

(Diane)

### Theme 2: psychosocial impact of perineal wound dehiscence

#### Subtheme: denial

Some of the women struggled to acknowledge the dehiscence, they felt that by ignoring the problem it would still be alright or even better that it would actually go away as Sue's account suggests below.

If you leave me alone, don't poke…it will go away type of thing…would have buried my head in the sand a bit really and left it. (Sue)

#### Subtheme: sense of failure or self-blame

Women relayed a sense of failure for not conforming to what they perceive as normality in day-to-day activities, including their feelings of ‘self-blame’ that they are in this situation. This was particularly evident for Diane as the following extract reveals.

Really thought bad about myself, my stitches coming undone made me feel really bad about myself … honestly it was … I really thought that bad of myself. (Diane)

#### Subtheme: fear

A sense of fear was expressed by nearly all women interviewed either related to their current experience or for future childbirth. Women spoke about being scared of having stitches again, petrified of giving birth next time and that the whole process was too much to go through again. Jenny, was currently pregnant for the second time and sadly, despite talking to her midwife about it, the extract below illustrates that Jenny remained fearful of childbirth.

I'm petrified, I'm petrified, I must admit…I'm just worried because this scar being still sore and it's still quite new, I'm worried about it popping open or making it difficult while I'm in labour, don't know … I don't know. I just keep going over these things, going around my head thinking I'm not going to be able to do it. Maybe I'm being stupid. I would have liked the opportunity to discuss delivery this time. I told my midwife what had happened, but she didn't say anything so I thought it'd be alright, but it's still a concern of mine. It's sort of like I'm petrified. (Jenny)

#### Subtheme: altered body image

Women also revealed the extent of their fears relating to an altered body image, with thoughts of being deformed.

My stitches (breaking down) that was the worst thing, because it's your body isn't it ….my heart dropped, I thought I was going to be deformed. I don't think they should just leave a massive hole down there to heal back by itself, because it's just devastating. (Diane)

It was a lot of worry…it's not nice to have an open wound down there… When I first had it I thought oh god, I was going to be deformed. (Jenny)

#### Subtheme: isolation

Most women spoke about the feeling of isolation and not being able to leave the house as soon as they had expected either as a result of the pain or feelings of anxiety and lacking in confidence.

### Theme 3: sexual impact of perineal wound dehiscence

This theme describes the impact of both resuming sexual intercourse following their perineal wound dehiscence and the long-term sexual morbidity that some women were still experiencing up to 9 months following childbirth. Women in both treatment groups reported issues related to sexual morbidity as the following brief extracts reveal.

#### Resutured group

Very painful, (responded immediately) worse following this baby. (Sue)

Wasn't good at first … I must admit, it was quite painful, even now the scar tissue is quite sore sometimes when I wipe myself, when I go to the toilet. (Jenny)

#### Expectancy group

At first it felt different because of the piece of tissue that was swollen. (Fiona)

First time was really scary, frightened about it just ripping apart and the pain. I was petrified and it did hurt, felt like bruising…asked him (referring to partner) to have a look, he was good like that, then I asked him how it felt … he'd wind me up for a bit, then he was like no it feels normal. (Cathy)

### Theme 4: satisfaction with healing

Theme 4 provides evidence from all of the women interviewed relating to their personal experiences of wound healing, a recurrent theme in the narratives.

#### Resutured

She (perineal care midwife) removed some stitches. (Sue)

Healed really well, after one or two had opened again…think I had gristle or something, (referring to over granulation tissue). (Diane)

It's still quite raised, so you can feel it. It's healed a lot better than I thought to be honest. (Jenny)

#### Expectancy

Looked in the mirror, looks weird…it's just like you can see where it was stitched and come unstitched. (Nicola)

Felt like my right hand side looked lower than my left …. I was fretting that, that it all dropped if you get me. I asked him (partner) can you just please look….he said it looks fine. (Cathy)

Looking back, would have been better to have it re-stitched straight away, it's taken a long time to heal and I thought at 7 months I'd have to go back to the beginning and have it re-stitched. (Fiona)

### Theme 5: participating in the RCT (a priori)

Theme 5 describes women's experiences of taking part in the PREVIEW RCT and encompasses their understanding of the randomisation process and how they felt about completing the trial questionnaires.

#### Subtheme: understanding the randomisation process

Despite the strong preference for treatment options apparent in the following interview extracts, women did appear to understand the concept of randomisation.

I was praying that I could be…they'd come back, that they'd stitch me. I was well happy when told I was going to be re-stitched and since I had it done, then that made a whole lot of difference. I did put it in the thing (referring to the questionnaire) that if I didn't have it done it would have made… played a big ….massive ….I don't know…I think I would have been really unhappy. (Diane re-suturing)

Diane did indeed make a comment on her questionnaire, following it with a smiley face, ☺ illustrating her contentment for being resutured.

Cathy, who revealed that she was extremely emotional when she attended the recruiting site for review of her dehisced perineal wound, also had a strong preference for a treatment option, only this time it was for expectancy:

When the midwife asked me about putting the numbers in and picking which one you do, I was like please, please, please, please come back with tablets because I can't … I don't want an epidural anyway. She then (referring to the midwife researcher), she was like, you might have to have this … I said, no, no, no, no… {laughs} then she come back in and I was like ‘I love you’ {laughs} when I was told I was in that group (referring to expectancy). (Cathy)

Apprehensions relating to being readmitted to hospital, with concerns for childcare and the likelihood of requiring regional anaesthesia for the resuturing were voiced by women who had a preference for expectancy.

#### Subtheme: completing the trial questionnaires

All women interviewed felt that the PREVIEW pilot RCT questionnaires^18^ were straightforward easy to complete and that they were not too long. None of the women felt that there were any additional questions to ask and they all reiterated that the study had addressed outcomes that were important to them. Free text annotated sections were acknowledged as an area which allowed the women to include any additional information that they considered important.

#### Subtheme: attending for hospital appointments

How women felt about attending for hospital appointments to assess wound healing is crucial for the planning of the definitive study to optimise follow-up rates. However, women's experiences can also demonstrate to commissioners the value of perineal care clinics in areas where these are not already established. The accounts below demonstrate that women felt reassured with the advice they were given, that continuity and familiarity of being seen by the same clinician was important to them and that the care they received was both sensitive and responsive to their individual needs.

It wasn't just all about the stitches if you know what I mean ….they (the midwife researchers) were going through things … asking me how I was in general. I felt comfortable around them … so because I mean, sometimes you think … I've got to take my knickers off again with someone else. (Jenny)

The midwife, she was brilliant, she come and got me first (waiting to be seen for trial eligibility). (Cathy)

She was an angel, (referring to the perineal care midwife) when I come to the hospital one day and just burst into tears, I sat there and cried because I was terrified. (Cathy)

#### Subtheme: positive and negative experiences of the RCT

Women's positive experiences were primarily focused on the fact that the study was taking place and receiving the preferred treatment allocation as the following extracts reveal:

I was so grateful I was picked, it was all excellent…honestly. (Diane)

Definitely the right decision. (Sue)

I was pleased for you to come back in and said I was allocated into that group. (Nicola)

Any questions I had were answered. (Jenny)

Only one of the women interviewed had a negative experience associated with the trial procedure of resuturing and this focused on delays waiting to be transferred to theatre. This situation occurred due to emergency procedures taking priority.

Sue expressed her discontentment:

I felt neglected and ignored, just sat there (waiting to be transferred to theatre) without anyone coming near and giving us any information. Just something, it was like being in prison and this little eight by eight cell, stuck there all day with no daytime telly with a new born baby. And not even two chairs, there was only one chair. So that was, I thought that whole process perhaps could have been dealt with a bit better really. (Sue)

Interestingly Sue was also able to turn a negative experience in to a more positive one, she explained that:

It felt like more messing about, but I knew it was the right thing to do. (Sue)

#### Subtheme: women's acceptability of the treatment options

Listening to six women's accounts of their treatment allocation and the positivity that encapsulates their experiences, the majority of them were happy with the treatment they received. Only one woman (Fiona) felt that perhaps resuturing may have been a better option for her from the outset as she had a particularly protracted period of healing.

## Discussion

A key objective for conducting this study was to explore women's unique experiences of a dehisced perineal wound especially as the literature suggests that women expect to return to normality almost immediately following childbirth.^13^ ^14^ When reality does not quite meet with these expectations, particularly with an unexpected morbidity such as a wound dehiscence there can be an incredible loss of self-esteem and sense of failure.^12^ ^39^

Therefore, underpinning the powerful words of Walsh^40^ who states, “that disregarding women's views and experiences when developing evidence-based clinical guidelines is regarded as not only an injustice to women, but an indictment of the professional care ethic”. The results of women's open and honest narratives which have the potential to inform future practice and guidance have been respected and authentically presented.

The physical impact of the dehisced wound was a recurrent theme from this study and encompassed infection as a subtheme. When women were asked what their main concern was when their wound had broken, all expressed anxieties about avoiding infection or preventing it from becoming worse. Not surprising given the study referred to previously that suggested that 1 in 10 women will sustain a wound infection following primary repair of perineal trauma.^4^ Women's narratives in this phase of PREVIEW are consistent with other studies both quantitative and qualitative that healing of perineal trauma wounds particularly avoiding infection and dehiscence is paramount to many women and their partners following childbirth.^17^ ^41–43^ Additionally, altered body images with thoughts of being deformed and in some circumstances accepting that the perineal area ‘looks different’ were also relived by several women interviewed for PREVIEW and support the findings of other published qualitative research relating to perineal trauma.^16^ ^17^ While there were some who felt and looked at their perineum's even asking their partners to look for reassurance of normality, other studies have reported that women that could not bear to look at it or touch the area.^16^

Perineal pain was a subtheme of the physical impact of wound dehiscence and in some respects this was anticipated, as perineal pain of varying intensity is experienced by the majority of women following vaginal delivery.^43–46^ What the researcher (LD) was not quite prepared for, demonstrated in reflective journal extracts was the emotive and powerful responses such as ‘horrific’ and ‘petrifying’ used by women to describe the intensity of their pain. Even the most complex of quantitative measures of pain could not emulate the poignancy expressed in the narratives of women's experiences of pain.

Satisfaction with wound healing was a recurrent theme from the qualitative study conducted with women who participated in the PREVIEW RCT. Most of the women interviewed were between 7 and 9 months following childbirth and their experiences suggest that the aesthetic results of wound healing extend beyond that of the 6 months outcome measure commonly associated with quantitative research investigating perineal trauma. In addition, women from both groups revealed accounts of being treated for over granulation tissue. One woman in the expectancy group also referred to a discussion between herself and a perineal care specialist midwife regarding the possible need for further surgery at a follow-up appointment some 7 months following childbirth. To her relief this was subsequently not required. The outcome was not quite the same for a woman in the study by Salmon^17^ who after 18 months of desperation with perineal morbidity was finally referred back to the hospital for perineal refashioning. Unfortunately for some women who experience a perineal wound infection and or dehiscence, particularly those that have been managed by expectancy, there is also the potential for further corrective surgery, perineal refashioning and excision of excessive scar tissue or other procedures associated with treating perineal dysfunction and altered body image.^46^ Indeed, 50% of the women who were managed by expectancy in the PREVIEW pilot RCT^18^ felt that their perineum was not back to normal, one may therefore postulate that the potential for further intervention is not an unrealistic assumption.

The psychosexual morbidity associated with poor healing and altered body image must not be underestimated, as this study demonstrates. Women interviewed from both treatment groups reported issues relating to sexual morbidity some 6–9 months after childbirth. They feared the unknown as intercourse was resumed, with an almost acceptance that it was going to feel different, having experienced childbirth, emulating the findings of other qualitative studies.^13^ For several women there was no association with previous sexual morbidity but for others there was, particularly for one woman who was still breast feeding, and whose other children were both under 5 years old. Discussing issues around sexual health and sexual morbidity can be uncomfortable, even somewhat embarrassing and the silence comes from new mothers as well as from some healthcare professionals too.^47–49^ This study revealed that when asked, all women were in fact willing to share their experiences of varying degrees of sexual morbidity following childbirth.

A further objective for this study was to establish women's experiences of participating in the pilot RCT^18^ crucial to ensure that a future study is as robust as possible. This resulted in the ‘a priori theme of ‘participating in the RCT’. Women interviewed were asked about their understanding of the randomisation process and the findings suggest that they were all aware that they would be allocated either resuturing or expectant management. However, nearly all of the women in the study had a strong preference for a treatment option and yet still consented to take part in the study. Sharing their incredibly emotive experiences as they waited in suspense of the randomisation allocation would lead a researcher to postulate that compliance may have been a potential issue if, by chance, they were not allocated their treatment preference. Indeed, in the pilot RCT, two of the women randomised to resuturing did not receive the intervention. Although this was attributed to anxiety surrounding the procedure in one case, it is possible that both women would have preferred expectancy.

For some women interviewed, their experience of childbirth and the vulnerability of that early postnatal period clearly demonstrates some of the reasons why recruitment into research studies at a particularly vulnerable time can prove challenging. Diane clearly recalled that she had a preference for resuturing. What was humbling in this case was that Diane was a young 20-year-old new mother, who had experienced an operative vaginal delivery with double application of instruments. Her baby daughter was then transferred to the neonatal intensive care unit for several days. Diane's perineal wound then dehisced leaving a ‘massive hole’ to use her own words and yet here she was, desperate to take part in the study so she had a chance of being resutured. An intervention that she clearly believed should be offered as a treatment option for women ‘so glad I was picked, it (resuturing) should be offered to most women’.

Diane's motivation to take part in the study was driven by the possibility of receiving an intervention that she actually wanted, but one that was currently only offered as part of a clinical trial. She also had a genuine desire to help other women by participating in the research. In fact, a degree of altruism was evident in all women in this qualitative study and supports similar motivational theories revealed in other studies.^50–52^

This qualitative study made additional attempts to establish if the intervention of resuturing is an acceptable treatment option for the women (a subtheme of participating in the pilot RCT). Women's experience of receiving the intervention of resuturing was extremely positive in the three women interviewed. Their accounts were supported by the free text comments in the pilot RCT questionnaires. The only negativity associated with the secondary resuturing was associated with the theatre delays and waiting to be transferred for the procedure. In contrast, retrospectively, more women who had received expectant management felt that they would have preferred to have been resutured. Although there were also reports from women who felt that expectancy had been the right approach for them.

Women's satisfaction with attending for trial appointments was a subtheme of participating in the pilot RCT. Women were extremely satisfied with their clinical appointments to follow-up wound healing with most women appreciative of the additional information and support they received. All women interviewed were from organisations where access to a perineal care clinic was available. However, women in some of the other recruiting organisations would not have received the level of follow-up unless they were in the PREVIEW study and several comments in the free text sections of the pilot RCT^18^ questionnaires reflected this.

There have been repeated recommendations that have stressed the need for perineal care clinics.^13^ ^15^ ^16^ ^45^ The value of dedicated multidisciplinary perineal care clinics are without doubt, unquestionable.^45^ Nationally, the PREVIEW pilot study has been catalyst for change in a number of units that have submitted successful business cases for the introduction of perineal care clinic. Providing much needed information, support and reassurance is an area that women have persistently expressed as lacking.^13^ ^14^ ^16^ Perineal care clinics can enable the provision of timely evidence-based, woman-centred care, which is sensitive and responsive to the needs of individual women at a time when they need it the most.

### Strengths and limitations

This nested qualitative phase of PREVIEW has complemented the data obtained from the pilot RCT^18^ adding as Bowling^53^ quite rightly acknowledges a degree of comprehensiveness and richness to the whole study, placing the quantitative data in meaningful social contexts. Addressing lessons learnt and knowledge gained is now pivotal to inform the design of the future definitive study and towards providing much needed answers towards the efficacy of the interventions. The results of each paradigm, which demonstrated that both treatment options are acceptable to women, are important to commissioners, healthcare providers and women for the successful commissioning and implementation of future local and national guidance.

Our study also highlighted several limitations for the reader to consider. We only interviewed RCT participants. Interviewing non-randomised women who fulfilled eligibility criteria could potentially have proved beneficial to the planning of the definitive study; however, this would have required additional ethics approval and incur additional time and finances that were not factored into the original research proposal or funding application.

Owing to geographical locations of the recruiting sites, five of the six women interviewed were from one organisation and therefore may not truly reflect women's experiences of participating in research in other sites. All women interviewed were also of white ethnicity. Potentially, responses of women from wider ethnic backgrounds may have revealed different views and hence, this needs to be factored in to the methodology of future research in this field.

The free text annotations in the pilot RCT questionnaires do however suggest that women's experiences of taking part in the RCT were very positive, demonstrating the benefits of the mixed methodological approach.

### Implications for clinical practice

Although resuturing may not be a favoured option by all women, for some there was an overwhelming desire for this treatment option. The authors acknowledge that further research is needed to provide robust evidence of the efficacy of this intervention. However, we would urge providers of maternity services to consider offering this as an alternative treatment option to expectant mothers’ management when clinically indicated and requested by women themselves. In addition, the authors would strongly recommend that maternity services consider the implementation of perineal care clinics and clear referral pathways, for women who experience perineal problems in the postnatal period, where these are not currently provided.

### Implications for future research

Conducting this qualitative study has highlighted that the main outcomes important to women were addressed in the pilot RCT and should continue to be evaluated in a future definitive study. Several areas to consider for future research, particularly given the strong patient preference for a treatment option, would be to consider the inclusion of a patient preference randomisation alongside a conventional RCT. Future research exploring women's experiences of perineal wound dehiscence should also aim to include women from various ethnic backgrounds and maternity organisations to strengthen the generalisability of research findings to a wider, more diverse population of women.

## Conclusion

This study is, to the best of our knowledge, the first research report exploring women's experiences of perineal wound dehiscence. Women have finally been given an opportunity to share with healthcare professionals and ultimately each other, their previously ‘unheard’ experiences of this unfortunate complication of childbirth. The powerful testimonies presented disclose the extent of morbidity experienced while also revealing a strong preference for a treatment option. Women have been given a voice in the assessment of the interventions which future women will receive and this can only serve but to enhance future practice and research.^54^
